# Topical tranexamic acid in intramedullary nailing for the treatment of intertrochanteric fractures in the elderly: A protocol for systematic review and meta-analysis

**DOI:** 10.1097/MD.0000000000032854

**Published:** 2023-02-10

**Authors:** Jiakai Zhang, Xiaoyuan Fan, Yi Zheng, Junlong Wu, Xinhua Yuan

**Affiliations:** a Department of Orthopedics, Ningbo No.2 Hospital, Ningbo, China; b Department of Gastroenterology, The Affiliated People’s Hospital of Ningbo University, Ningbo, China.

**Keywords:** intertrochanteric fracture, topical application, tranexamic acid

## Abstract

Numerous studies have demonstrated that the use of tranexamic acid (TXA) intravenously minimizes bleeding, lowers transfusion rates, and does not raise the risk of complications during major orthopedic surgery. Concerning the effectiveness of the topical application, there are, nevertheless, inconsistent findings. We aimed to develop a protocol for systematic review and meta-analysis on the benefits and safety of topical TXA in intramedullary nailing for the treatment of intertrochanteric fractures in the elderly.

**Methods:**

PubMed, Embase, and the Cochrane Library will all be searched for randomized controlled trials published from the database inception to October 15, 2022. The primary outcomes will be intraoperative blood loss, hidden blood loss, total blood loss, transfusion rate, transfusion units, operative time, thromboembolic events, and mortality. The risk of bias will be evaluated using the Cochrane risk of bias assessment tool. Review Manager 5.3 will be used for the analysis.

**Results:**

The effects and safety of topical TXA in intramedullary nailing for the treatment of intertrochanteric fractures in the elderly will be quantified in this study.

**Conclusions:**

The study’s findings will assist doctors in determining if topical TXA use is secure and efficient.

## 1. Introduction

About half of the hip fractures in older people are femoral intertrochanteric fractures, which are typical hip fractures.^[1]^ Its prevalence is predicted to reach 6.3 million annually in 2050 as the population ages.^[2]^ Today, surgery is typically advised since conservative therapy might have a 30% mortality rate within a year and frequently results in lung infections, decubitus ulcers, and deep vein thrombosis.^[1,3]^ Intertrochanteric fractures frequently result in significant bleeding at the end of the fracture, leaving the patient anemic before surgery.^[4]^ The prognosis may deteriorate due to anemia. Allogeneic blood transfusions are frequently necessary for patients with intertrochanteric fractures, which can lead to problems such as transfusion reactions and infections.^[4,5]^ Thus, minimizing perioperative blood loss is essential to minimizing complications and enhancing prognosis.^[5]^ The most preferred surgical management currently is intramedullary fixation, which is minimally invasive, reliable, and has low intraoperative blood loss (IBL).^[6–8]^ Although IBL is minimal, hidden blood loss (HBL), which is far more common following intertrochanteric fractures, has been observed to be much higher.^[4,8]^ As a result, lowering HBL has become crucial to patients’ recovery.

Tranexamic acid (TXA) is a synthetic antifibrinolytic drug.^[9]^ Many studies have confirmed the effectiveness of the intravenous application of TXA in hip and knee replacement in reducing bleeding and decreasing transfusion rates without increasing the risk of complications.^[10,11]^ Similar efficacy has been reported in minimally invasive intramedullary nailing for intertrochanteric fractures.^[12–14]^ Although topical administration of TXA has only occasionally been reported, some studies have shown that it is safe and effective,^[15,16]^ whereas others have found the contrary.^[17,18]^ The efficacy and safety of topical application are still debatable.

Therefore, we will summarize the currently available randomized controlled trials in this systematic review and meta-analysis to evaluate the safety and efficacy of the topical application of TXA in intramedullary fixation for the treatment of intertrochanteric fractures in the elderly.

## 2. Methods

The Preferred Reporting Items for Systematic Reviews and Meta-Analysis Protocols declaration has been followed by this protocol. Our International Prospective Register of Systematic Reviews registration number for this protocol is CRD42023380352.

### 2.1. Search strategy

The electronic databases of PubMed, Embase, and the Cochrane Library will be searched from the inception to October 15, 2022. Only English studies will be included. Two reviewers will independently conduct literature searches using the following keywords: “tranexamic acid,” “TXA,” “topical application,” “intertrochanteric fracture,” “peritrochanteric fracture,” “hip fracture,” “intramedullary nail,” “IMN,” “proximal femoral nail anti-rotation,” “PFNA,” and “randomized controlled trials.” Additionally, a manual search of the references of previously published literature will be conducted to find any other studies that qualify.

### 2.2. Inclusion criteria

1. Individuals receiving proximal femoral intramedullary nail surgery for intertrochanteric fracture.
2. Age ≥ 65 years old.
3. Topical application of TXA in the experimental group.
4. No TXA administration in the control group, with placebo or saline.
5. Only randomized controlled trials will be included.
6. The clinical outcome includes the following: IBL, HBL, total blood loss (TBL), transfusion rate, transfusion units, operating time, and thromboembolic events, including symptomatic deep vein thrombosis, pulmonary embolism, myocardial infarctions, and strokes, as well as mortality. At least 1 clinical outcome data must be included in the trials.

### 2.3. Exclusion criteria

1. Patients with open or multiple fractures.
2. Full text is not available.
3. Studies with insufficient clinical outcome data.

### 2.4. Study selection and data extraction

Two reviewers will independently extract data according to the inclusion and exclusion criteria. Any disagreement on data extraction will be resolved by the third reviewer. The demographic characteristics will extract as follows: first author, year of publication, region, patients, age, gender, fixation method, thromboprophylaxis drugs, interventions, transfusion criteria, operative time, IBL, HBL, TBL, transfusion rate, transfusion units, follow-up, thromboembolic events, and mortality.

### 2.5. Risk of bias assessment

Two investigators will separately assess the risk of bias of the included studies using the Cochrane risk of bias assessment tool. Discrepancies will be resolved by discussion or consultation with the third reviewer.

### 2.6. Statistical analysis

The Cochrane Collaboration’s Review Manager 5.3 (Update Software Ltd, Oxford, Oxon, UK) will be utilized for the analysis. *P* < .05 is considered statistically significant. Relative risk and 95% confidence interval will be used for dichotomous outcomes; the mean difference and 95% confidence interval will be used for continuous outcomes. Statistical heterogeneity for all enrolled studies will be evaluated using the *χ*^2^ test and *I*^2^ statistic. Statistical heterogeneity will be classified into 3 categories: high (*I*^2^ ≥ 50%), moderate (25% ≤ *I*^2^ < 50%), and low (*I*^2^ < 25%). If the *P* value of heterogeneity is <0.1, heterogeneity will exist. The randomized-effects model will be performed when *I*^2^ ≥ 50%, Otherwise, the fixed-effects model will be chosen.

### 2.7. Sensitivity analysis

We will conduct a sensitivity analysis by including only studies with a low risk of bias to see if the results are consistent under different assumptions.

### 2.8. Ethics

This study does not require ethical approval because it is a secondary study.

## 3. Discussion

The use of TXA in hip fractures has been the subject of several recent meta-analyses.^[19,20]^ TXA was discovered to be beneficial when administered intravenously in lowering IBL, HBL, and TBL. It was also found to decrease transfusion rates and volumes without increasing operating time, thrombotic events, or mortality.^[10–14]^ However, the conclusions remain debatable due to the various TXA administration methods, dosages, and frequencies, as well as the various surgical strategies and outcome assessments.^[19,20]^ The literature is sparse on the local application of TXA. There is no relevant meta-analysis. This is the first systematic review and meta-analysis to focus on the local application of TXA and intramedullary nailing in the treatment of intertrochanteric fractures in the elderly. We believe that the study’s findings will reveal the efficacy and safety of the topical application of TXA.

## Author contributions

**Conceptualization:** Jiakai Zhang, Junlong Wu.

**Data curation:** Xiaoyuan Fan, Junlong Wu.

**Formal analysis:** Jiakai Zhang, Xiaoyuan Fan.

**Funding acquisition:** Xinhua Yuan.

**Investigation:** Yi Zheng.

**Methodology:** Yi Zheng, Xinhua Yuan.

**Project administration:** Jiakai Zhang, Junlong Wu, Xinhua Yuan.

**Supervision:** Xinhua Yuan.

**Validation:** Xinhua Yuan.

**Writing – original draft:** Jiakai Zhang.

**Writing – review & editing:** Xinhua Yuan.
